# Breaking the cycle: how poverty affects children’s oral health in developing nations—a rapid review

**DOI:** 10.3389/fpubh.2025.1612026

**Published:** 2025-09-17

**Authors:** Arlette Suzy Setiawan, Ratna Indriyanti, Susi Sukmasari

**Affiliations:** ^1^Department of Pediatric Dentistry, Faculty of Dentistry, Universitas Padjadjaran, Bandung, Indonesia; ^2^Department of Pediatric Dentistry and Public Health, Kulliyah of Dentistry, International Islamic University Malaysia, Selayang, Malaysia

**Keywords:** oral health status, children, socioeconomic factors, poverty, developing countries

## Abstract

**Background:**

Oral diseases remain a significant public health challenge worldwide, disproportionately impacting children in developing countries due to socioeconomic hardship and limited healthcare access.

**Purpose:**

This rapid review evaluates the relationship between low household income and children’s oral health outcomes in developing countries.

**Method:**

We followed PRISMA 2020 and Cochrane Rapid Review guidelines. A systematic search of five databases (2012–2022) using refined keywords identified eligible studies. Quality assessment used the NIH tool.

**Results:**

Of 1,574 articles identified, 13 met inclusion criteria. Most were cross-sectional studies from China, India, Nigeria, Brazil, and Syria. A consistent association was found between lower socioeconomic status and worse oral health outcomes, including higher DMFT/dmft scores, gingivitis, and early childhood caries (ECC).

**Conclusion:**

Low socioeconomic status (SES) is associated with worse oral health outcomes in children in developing countries.

## 1 Introduction

Oral and dental health was a problem for nearly 3.5 billion people worldwide in 2020. Untreated dental caries in permanent teeth remain the most prevalent health condition globally, affecting an estimated 2.5 billion people. Severe periodontal disease affects approximately 10.8% of the global population. In addition, lip and oral cavity cancers rank among the 15 most common cancers worldwide (1). Untreated oral diseases can cause pain, infection, and reduced quality of life and productivity. Good oral health positively impacts nutrition, employment, self-confidence, and social participation (2).

Lower socioeconomic status is strongly linked to poorer oral health outcomes (2). Previous studies in developed countries have shown that low-income people have worse oral health compared to those with high-incomes (3). Economic inequalities affect healthcare access and utilization, influencing oral health outcomes (4). The link between economic factors and dental and oral health is reflected in the increasing prevalence of oral health problems among populations in low-income countries (2). Economic inequalities within the population continue to influence individuals’ decisions when healthcare facilities and services are chosen, ultimately affecting the outcomes of the care they receive (4).

According to the World Health Organization (WHO), oral health problems are more prevalent in low and middle-income countries (LMICs), with three-quarters of affected individuals residing in these regions (2). WHO indicates that developing nations endure a considerable burden of oral disorders, including dental caries, advanced periodontal disease, tooth loss, and oral malignancies. The WHO indicates that more than 45% of the global population is afflicted by oral disorders, with three-quarters of these individuals living in low- and middle-income nations (5).

Comprehensive data on the oral health of children from low-income households in developing countries are scarce, posing challenges for developing targeted interventions (2). Most existing literature focuses on adults in developed countries, leaving a research gap regarding children in developing nations. Understanding the relationship between poverty and oral health in childhood—a crucial phase for establishing lifelong habits—is essential. This rapid review aims to synthesize current evidence on how low household income influences children’s oral health outcomes in developing countries.

## 2 Method

This rapid review was conducted in accordance with Cochrane Rapid Review guidelines and PRISMA 2020. We used the PICO framework as follows: – Population: Children (0–18 years) from low-income families in developing countries – Intervention: Assessment of oral health status using indices (DMFT/ dmft, GI, OHI-S) – Comparison: Children from higher-income households – Outcome: Prevalence of caries and severity, ECC, gingivitis, and oral hygiene.

The research question was: “What is the impact of poverty on oral health status of children in developing countries?”.

We searched ScienceDirect, PubMed, Scopus, Web of Science, and Livivo using terms including “oral health,” “dental health survey,” “oral health status,” “socioeconomic factors,” and “children.” The term “low socioeconomic status” was not included as a primary keyword to avoid overly restrictive results and ensure broader retrieval. Boolean logic was adapted for each database (detailed in Table 1).

**Table 1:** Summary of findings from 9 selected articles based on data eligibility criteria.

No	Author, Year, and Title	Location	Design and population (*n*)	Assessment	Result	Conclusion	Risk bias assessment
1	Agbaje H. O et al. (2016) (23) Digit Sucking, Age, Sex, and Socioeconomic Status as Determinants of Oral Hygiene Status and Gingival Health of Children in Suburban Nigeria.	Ife Central Local Government Area, Osun State, Nigeria.	Cross Sectional *n* = 345 Pre-school and school children aged 1–12 years-old	Oral hygiene status Oral Hygiene Status with OHI-S (Oral Hygiene Index Simplified) by Greene and Vermillion (Baik/Good (0.0–1.2), Sedang/Fair (1.3–3.0), Buruk/Poor (>3.0)). Gingivitis severity with Gingival Index (GI) by Loe and Silness (Ringan/Mild (0.1–1), sedang/ moderate (1.1–2), parah/ severe (2.1–3)).	Oral and Dental Hygiene Status 55% of children have good oral hygiene. 42.6% of children have fair oral and dental hygiene. 2.3% of children have poor oral and dental hygiene. Children aged 6–12 years (AOR: 0.26; 95% CI: 0.20–0.35; *p* < 0.001) have poorer oral and dental hygiene compared to the 1–5 year age group. Children from low (AOR: 1.37; 95% CI: 0.98–1.90; *p* = 0.06) and middle (AOR: 1.10; 95% CI: 0.77–1.56; *p* = 0.60) socio-economic status have poorer oral and dental hygiene compared to children from higher socio-economic backgrounds. Severity of Gingivitis: 82.9% of children aged 1–12 years have mild gingivitis, 16.9% have moderate gingivitis, and the remaining 0.2% have severe gingivitis. Children aged 1–5 years have a lower severity of gingivitis compared to those aged 6–12 years (AOR: 0.21; 95% CI: 0.14–0.31; *p* < 0.001). Children from low socio-economic status have higher gingival index (GI) scores than those from higher socio-economic backgrounds (AOR: 2.09; 95% CI: 1.32–3.31; *p* = 0.002).	A correlation exists between age and the severity of gingivitis as well as the dental hygiene status of children. Children between the ages of 6 and 12 exhibit an increased susceptibility to gingivitis and inferior oral hygiene relative to those aged 1 to 5 years. A notable correlation was identified between gingivitis and the family’s socio-economic level. Children from disadvantaged socio-economic circumstances face an elevated chance of getting gingivitis. This study did not address the fundamental explanations of the association between socio-economic position and gingival health.	Good
2	Zhang Jialan et al. (2021) (11) Association between socioeconomic status and dental caries among Chinese preschool children: a cross-sectional national study	China	Cross Sectional *n* = 40.360 Anak berusia 3–5 tahun	Prevalence of Dental Caries Based on dmft Index.	A substantial correlation exists between dental caries and both parental education and family income (*p* < 0.001). The incidence of dental caries in children is 62.5%, accompanied by an average dmft score of 3.35 ± 0.02. Children of parents with poor educational attainment and poverty exhibit elevated dmft scores (IRR 1.16; 95% CI: 1.10 to 1.23) relative to those from higher-income families.	There is a significant association between socio-economic status and both the prevalence of dental caries and dmft scores. Children from low-income families have higher dmft scores compared to those from higher-income households.	Good
3	Jindal et al. (2020) (12) Dental Caries in Relation to Socioeconomic Factors of 6 and 12-year-old Schoolchildren of Paonta Sahib, Himachal Pradesh, India: An Epidemiological Study	Paonta Sahib, Himachal Pradesh, India	Cross sectional n = 1,004 Children Aged 6 and 12 Years 437 children aged 6 years 567 children aged 12 years	Prevalence of Dental Caries Based on WHO 2013 DMFT Criteria	Prevalence of Dental Caries: A total of 582 children aged 6 and 12 years were found to have dental caries, with an overall prevalence of 58.0%. The prevalence was 63.6% among 6-year-olds and 53.6% among 12-year-olds. DMFT Scores: The average DMFT score was higher among children from low-income families (1.60) compared to those from middle-income (1.26) and high-income families (1.20). Dental Caries and Socio-Economic Status: Among the 582 children with dental caries, 25.4% were from the upper-lower class, 32.3% from the lower-middle class, 38.1% from the upper-middle class, and 4% from the upper class.	There is a relationship between the prevalence of dental caries and the socio-economic status of the family. Children from lower-income families experience a higher frequency of tooth decay compared to those from higher-income families.	Good
4	Sharma S. et al. (2013) (13) Oral Health Status of 9 to 12 year old school going children in Urban Meerut	Urban area, Multan Nagar, Meerut, India.	Cross sectional n = 534 Children aged 9–12 years	Oral Health Status Based on WHO Oral Health Surveys Method Oral hygiene status assessed using the Simplified Oral Hygiene Index (OHI-S). Dental caries assessed using the DMFT index.	Oral Hygiene: The prevalence of oral hygiene status showed that 54% of the population had fair oral hygiene, 34.3% had good oral hygiene, and 12% had poor oral hygiene. Dental Caries: The average DMFT score for permanent teeth was 0.89, with the decayed component (D) contributing the most at 0.87. Association with Socio-Economic Status: Children from low-income families had a higher DMFT score (1.35) compared to those from high-income families (0.55). Gingivitis: A total of 53.4% of children had gingivitis, with moderate gingivitis accounting for 44.4%, mild gingivitis for 7.9%, and only 1.1% experiencing severe gingivitis.	There is a significant association between socio-economic status and dental caries. DMFT scores increase progressively as a child’s socio-economic status decreases.	Fair
5	Paula Glaucia Maria., et al. (2015) (15) The impact of social determinants on schoolchildren’s oral health in Brazil.	Juiz de For a, Minas Gerais, Brazil.	Cross sectional n = 515 Children aged 12 years	Dental Caries Based on the DMFT Index	Dental Caries: The DMFT score was 1.09, with 61.2% of the population having no caries experience (DMFT = 0), and 38.8% having a DMFT score greater than 0. Socio-Economic Status: Children from low-income families were 1.89 times more likely to have caries experience.	Socio-economic factors have the most significant association with caries experience among 12-year-old schoolchildren. The DMFT score of children in Juiz de Fora is lower than the national average DMFT score of children in Brazil.	Fair
6	Liu, Mingshan., et al. (2022) (21) Early childhood caries prevalence and associated factors among preschoolers aged 3–5 years in Xiangyun, China	Xiangyun, China.	Cross sectional n = 1,280 Children aged 3–5 years	Early Childhood Caries (ECC) Based on the WHO 2013 dmft Index	Early Childhood Caries (ECC): A total of 74.3% of children aged 3–5 years experienced ECC, with an average dmft score of 4.9 ± 5.0. The prevalence was 64.9% at age 3, 71.5% at age 4, and 80% at age 5. Socio-Economic Status: The prevalence of ECC was significantly lower (66.4%) among children from high-income families compared to those from low-income families (76.6%) (*p* = 0.006).	There is a significant association between low family income and children’s oral health status. A correlation exists between monthly family income and the risk of ECC. Children from low-income families have a significantly higher prevalence of ECC compared to those from high-income families.	Good
7	Li., et al. (2020) (19) The status and associated factors of early childhood caries among 3- to 5-year-old children in Guangdong, Southern China: a provincial cross-sectional survey.	Guangdong, Southern China.	Cross sectional *n* = 2,592 Kindergarten children aged 3–5 years	Early Childhood Caries (ECC) based on the WHO 2013 dmft Index.	Prevalence of Early Childhood Caries (ECC): 68.3% of the population experienced ECC, with an average dmft score of 4.36. The prevalence of ECC was 58.2% among 3-year-olds, 68.4% among 4-year-olds, and 78.4% among 5-year-olds. Socio-Economic Status: Children from low-income families had a higher ECC prevalence and dmft score (77.6%; 5.7) compared to children from high-income families (58.5%; 3.0).	There is a significant association between income level and the development of Early Childhood Caries (ECC).	Good
8	Alhaffar, et al. (2019) (14) Oral health and socio-economic status among children during Syrian crisis: a cross-sectional study	Damaskus, Syiria.	Cross sectional *n* = 811 Schoolchildren with an average age of 12 years	Dental Caries based on the WHO 2013 DMFT Index.	Dental Caries: The average DMFT score in the population was 3.36, with 86% of children having at least one tooth decayed, missing, or filled due to caries. Socio-Economic Status: Based on monthly income, the average DMFT scores among children were 5.65 for low-income families, 3.85 for middle-income families, and 2.43 for high-income families.	There is a significant association between children’s oral health status and socio-economic conditions. Children from low-income families have higher DMFT scores, indicating that they have poorer oral health status compared to children from higher-income families.	Good
9	Guan Y., et al. (2015) (20) Socioeconomic inequalities in dental caries among 5-year-olds in four Chinese provinces	Guangxi, Hubei, Jilin and Shanxi in China.	Cross sectional n = 1732 Kindergarten children aged 5 years	Dental Caries based on the dmft index.	Dental Caries: The average dmft score among children from low-income families (4.70) is higher compared to those from high-income families (2.63).	There is a significant association between monthly family income and dental caries in this study.	Good

Articles published between January 2012 and August 2022, in English or Indonesian, were included. Dissertations, gray literature, and non-full-text papers were excluded. Screening was conducted independently by three reviewers. Quality appraisal used the NIH tool (6).

Duplicate articles were removed, and the remaining articles were checked on the basis of the relevance of the title and abstract and then selected on the basis of full-text availability and content consistency with the study objectives. Further screening was performed by checking the study location to ensure that the studies were conducted in developing countries according to the World Economic Situation and Prospects 2020 classification (7).

**Figure 1:**
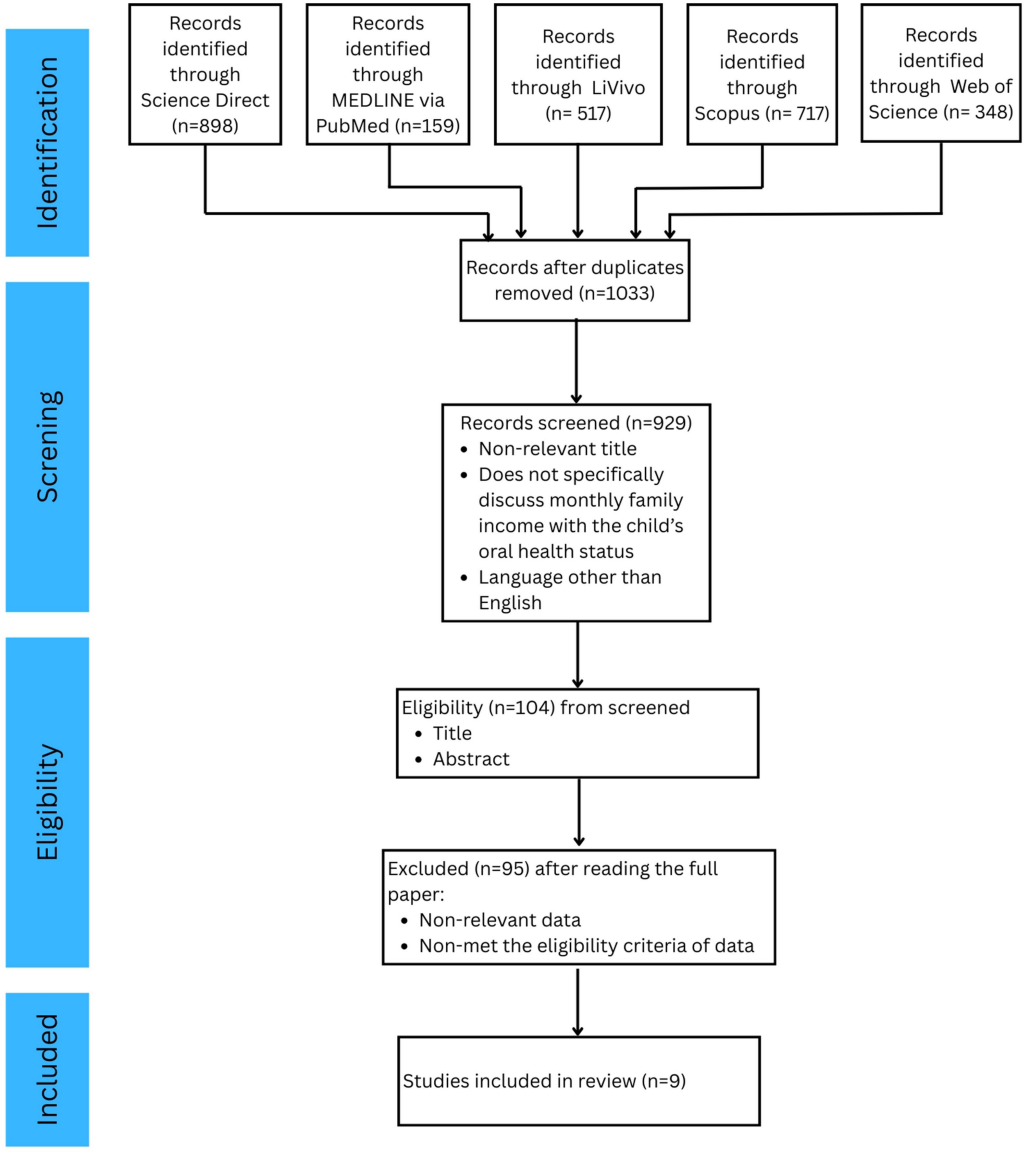
PRISMA flow diagram for the literature search.

The results of the expedited review process, including identification, screening, eligibility evaluation, and final selection, are illustrated in the PRISMA flow diagram (Figure 1). A preliminary search employing specified keywords and filters for publication years ranging from 2012–2022 yielded 1,574 articles across three databases. Upon eliminating duplicates, 1,033 articles remained.

The remaining publications were subjected to title and abstract screening according to the inclusion criteria, which included relevancy, language (English or Indonesian), and study design. This procedure produced 104 articles for comprehensive assessment. After a comprehensive evaluation of the complete texts, 95 papers were removed due to factors such as irrelevant demographics, settings not located in developing nations, or incompatible study designs. As a result, nine papers were incorporated into the final synthesis.

## 3 Result

Following screening, 9 articles were included. Most were cross-sectional studies, with no randomized or interventional designs. Countries represented included China, India, Nigeria, Brazil, and Syria. Findings consistently showed that children from lower-income families had higher rates of: – Dental caries (via DMFT/dmft index): higher prevalence and severity – Early childhood caries: more frequent in low-SES families – Gingivitis: elevated GI scores among disadvantaged children – Oral hygiene: more poor/fair hygiene linked to economic disadvantage. Additional factors influencing outcomes included: – Parental education – Sugar consumption – Tooth brushing frequency – Access to dental services. Table 1 summarizes key findings. Table 2 reports the risk of bias assessment.

**Table 2:** Risk of bias result.

No.	Criteria	Article																	
		1		2		3		4		5		6		7		8		9	
		Rater 1	Rater 2	Rater 1	Rater 2	Rater 1	Rater 2	Rater 1	Rater 2	Rater 1	Rater 2	Rater 1	Rater 2	Rater 1	Rater 2	Rater 1	Rater 2	Rater 1	Rater 2
1	Was the research question or objective in this paper clearly stated?	Yes	Yes	Yes	Yes	Yes	Yes	Yes	Yes	Yes	Yes	Yes	Yes	Yes	Yes	Yes	Yes	Yes	Yes
2	Was the study population clearly specified and defined?	Yes	Yes	Yes	Yes	Yes	Yes	Yes	Yes	Yes	Yes	Yes	Yes	Yes	Yes	Yes	Yes	Yes	Yes
3	Was the participation rate of eligible persons at least 50%?	Yes	Yes	Yes	Yes	Yes	Yes	Yes	Yes	Yes	Yes	Yes	Yes	Yes	Yes	Yes	Yes	Yes	Yes
4	Were all the subjects selected or recruited from the same or similar populations (including the same time period)? Were inclusion and exclusion criteria for being in the study prespecified and applied uniformly to all participants?	Yes	Yes	No	No	Yes	Yes	No	No	No	No	Yes	Yes	Yes	Yes	Yes	Yes	Yes	Yes
5	Was a sample size justification, power description, or variance and effect estimates provided?	Yes	Yes	Yes	Yes	Yes	Yes	Yes	Yes	Yes	Yes	Yes	Yes	Yes	Yes	Yes	Yes	Yes	Yes
6	For the analyses in this paper, were the exposure(s) of interest measured prior to the outcome(s) being measured?	Yes	Yes	Yes	Yes	Yes	Yes	Yes	Yes	Yes	Yes	Yes	Yes	Yes	Yes	Yes	Yes	Yes	Yes
7	Was the timeframe sufficient so that one could reasonably expect to see an association between exposure and outcome if it existed?	Yes	Yes	No	No	No	No	No	No	No	No	Yes	Yes	Yes	Yes	Yes	Yes	Yes	Yes
8	For exposures that can vary in amount or level, did the study examine different levels of the exposure as related to the outcome (e.g., categories of exposure, or exposure measured as continuous variable)?	Yes	Yes	Yes	Yes	Yes	Yes	No	No	No	No	Yes	Yes	Yes	Yes	No	No	No	No
9	Were the exposure measures (independent variables) clearly defined, valid, reliable, and implemented consistently across all study participants?	Yes	Yes	Yes	Yes	Yes	Yes	Yes	Yes	No	No	Yes	Yes	Yes	Yes	Yes	Yes	Yes	Yes
10	Was the exposure(s) assessed more than once over time?	No	No	Yes	Yes	No	No	No	No	No	No	Yes	Yes	Yes	Yes	Yes	Yes	Yes	Yes
11	Were the outcome measures (dependent variables) clearly defined, valid, reliable, and implemented consistently across all study participants?	Yes	Yes	Yes	Yes	Yes	Yes	No	No	No	No	No	No	Yes	Yes	Yes	Yes	Yes	Yes
12	Were the outcome assessors blinded to the exposure status of participants?	Yes	Yes	Yes	Yes	Yes	Yes	Yes	Yes	Yes	Yes	Yes	Yes	Yes	Yes	Yes	Yes	Yes	Yes
13	Was loss to follow-up after baseline 20% or less?	Yes	Yes	Yes	Yes	Yes	Yes	Yes	Yes	Yes	Yes	Yes	Yes	Yes	Yes	Yes	Yes	Yes	Yes
14	Were key potential confounding variables measured and adjusted statistically for their impact on the relationship between exposure(s) and outcome(s)?	Yes	Yes	No	No	No	No	Yes	Yes	Yes	Yes	Yes	Yes	Yes	Yes	Yes	Yes	Yes	Yes
Number of “Yes”		13	13	11	11	11	11	9	9	8	8	13	13	14	14	13	13	13	13
Conclusion		Good		Good		Good		Fair		Fair		Good		Good		Good		Good	

## 4 Discussion

This review highlights the persistent oral health gap between low- and high-income populations in developing countries. Despite methodological variation, all included studies support a strong association between poverty and poor dental outcomes.

Heterogeneity was observed in: – Age groups studied (1–5, 6–12, and 12 + years) – Oral health indices used – Definitions of income categories. Confounding variables—such as parental education, brushing habits, and sugar intake—were not consistently adjusted for, limiting causal inference.

All studies were observational. The lack of interventional or longitudinal data restricts conclusions about causality. Future studies should include these designs to assess the effectiveness of targeted public health intervention.

Socioeconomic factors, especially income level, play important roles in the dental and oral health status of individuals. The results revealed that people in low-income groups tend to have worse dental and oral health than those in high-income groups (8). This study revealed that oral health problems among children aged 0–18 years in developing countries included dental caries, early childhood caries, poor oral hygiene, and gingivitis are associated with low monthly household income.

### 4.1 Dental caries

A commonly used parameter for caries assessment is the decayed, missing, filled teeth (DMFT or dmft) index, which calculates the total number of decayed (D), missing (M), and filled teeth (F) (9). According to the World Health Organization (WHO), the DMFT index is primarily used to assess caries in permanent teeth, particularly among 12-year-old children, as a global indicator. The WHO classification includes five categories: very low (<1.2), low (1.2–2.6), medium (2.7–4.4), high (4.5–6.5), and very high (>6.5). However, this classification does not apply to primary teeth (dmft index), and there is no standardized WHO threshold for interpreting dmft scores across age groups. Therefore, caution is required when comparing caries severity across populations with different dentitions (10).

Five studies conducted in China (11), India (12, 13), Syiria (14), and Brazil (15) revealed a significant association between low socioeconomic status and high caries prevalence and severity in children from low-income families in developing countries. This association was demonstrated by the fact that children from low-income families had higher mean DMFT/dmft values than did children from high-income families. Studies conducted in China (3–5-year-old children) (11), India (12, 13) (6-, 9–12-, and 12-year-old children), and Syiria (14) (12-year-old children) have shown that more than 50% of the study population had caries. The dmft value in the Chinese study was 3.35 (11), whereas the DMFT values in the Indian study were 1.60 (12) and 1.35 (13), respectively, and the DMFT value in the Syrian study was 5.65 (14). These results support the impact of low family income on the prevalence and severity of caries among children in developing countries.

A Brazilian study of 12-year-old children reported a dental caries prevalence of 38.8%, which appears lower compared to the reported rates in studies from China, India, and Syria. However, direct comparison should be made with caution, as the Brazilian study focused on permanent dentition in adolescents, while the others primarily examined primary dentition in younger children. Differences in age, dentition type (DMFT vs. dmft), and epidemiological thresholds limit the comparability of these findings. Hence, it is more appropriate to compare studies with similar populations and dentition types. Nevertheless, among the 38.8% of children with dental caries, the risk of dental caries among children from low-income families was 1.89 times greater than that among children from high-income families (15).

The results of these five articles are consistent with those of previous studies showing that children from low-income families are more likely to experience severe toothaches and tooth decay (9). This finding supports the link between socioeconomic status and oral health and highlights that children from economically disadvantaged families are more likely to suffer from dental disease (9).

### 4.2 Early childhood caries

Early childhood caries (ECC) is a common oral health problem with a high prevalence in most countries (16, 17). This problem is more common in developing countries than in developed countries (18). The prevalence of ECC is usually assessed via the dmft index, which is similar to the method of assessing caries via the Caries Experience Classification established by the World Health Organization (WHO) in 2013 (10).

Multiple (multifactorial) factors contribute to the occurrence of early childhood caries (ECC), of which socioeconomic status is one of the most important influencing factors (16). A study conducted in Guangdong, China, reported that the prevalence of ECC in children aged 3–5 years was 68.3%. The overall mean dmft score was 3.72 ± 4.14, with children from families earning less than 3,000 yuan/month showing a higher mean dmft score (4.79 ± 4.49) compared to those from higher-income families (≥10,000 yuan/month) with a mean score of 2.34 ± 3.16 (19).

Another study conducted in four provinces in China (Guangxi, Hubei, Jilin, and Shanxi) reported similar results. The level of caries experience in this population was reflected in the dmft values of different monthly income groups. Children from low-income families had a dmft score of 3.29, children from middle-income families had a dmft score of 4.70, and children from high-income families had a lower DMFT score of 2.63 (20). A study in Xiangyun, China, also revealed a high prevalence of ECC in children aged 3–5 years (74.3%), with a dmft score of 4.9. Children from high-income families had a significantly lower prevalence of ECC (66.4%) than did those from low-income families (76.6%) (21).

These three studies conducted in China consistently revealed a significant association between oral health and socioeconomic inequality (19–21). The mean dmft scores among low-income children were significantly higher than in those from high-income families, suggesting a considerable caries burden in preschool-aged children.

The high prevalence of ECC in children from low-income families is closely related to the lack of awareness of the importance of healthy primary teeth. This lack of awareness remains a serious problem and further limits the access of families with lower socioeconomic status to optimal dentistry and dental care (18).

### 4.3 Gingivitis

Gingivitis is usually assessed via the gingival index (GI), which is considered a reliable indicator of the severity and extent of gingivitis (22). One study examined gingivitis in children aged 1–12 years in Nigeria and reported the prevalence of gingivitis in 983 children. The results revealed that 82.9% (815 children) had mild gingivitis, 16.9% had moderate gingivitis, and 0.2% had severe gingivitis. According to socioeconomic status, 75.5% of children from 21 low-income families had mild gingivitis, 24.1% had moderate gingivitis, and 0.3% had severe gingivitis (23).

A similar study in India reported that 53.4% of children had gingivitis. Among these patients, 44.4% had moderate gingivitis, and only 1.1% had severe gingivitis. The study also revealed that gingivitis was more common in children from low-income families than in children from high-income families (13).

### 4.4 Oral hygiene

A study in Nigeria examining children aged 1–12 years found no significant association between oral hygiene status and socioeconomic status (23). Another study in India on children aged 9–12 years reported varying levels of oral hygiene and noted that behavioral factors such as brushing frequency and sugar intake played a role (13). These findings highlight that oral hygiene may be influenced more by behavior than by socioeconomic status in some contexts.

### 4.5 Other factors influencing children’s dental and oral health

Other factors that influence children’s oral health include differences in parents’ education level (11, 14, 15), tooth brushing habits (19–21, 23), the consumption of sugary foods (12, 19, 20), and regular dental checkups (11, 14, 15, 19–21).

### 4.6 Limitations

The study included only publications in English or Indonesian. Gray literature, dissertations, and non-peer-reviewed studies were excluded. Most included studies were cross-sectional and limited in confounding control. No subgroup analysis by age group was conducted; future studies should stratify by developmental stage. The search strategy employed a combination of terms related to socioeconomic status and oral health to ensure comprehensive coverage of relevant literature. To balance specificity and inclusiveness, both general and specific terms related to poverty and oral health in children were incorporated.

## 5 Conclusion

This review consolidates evidence that low socioeconomic status (SES) is associated with poorer oral health in children in developing countries. Efforts to improve oral health equity should: – Prioritize early interventions in schools and community programs – Incorporate oral health into broader poverty-alleviation policies – Emphasize parent-focused education and access to preventive services.

Future research should adopt interventional or longitudinal designs, expand language and source inclusivity, and focus on age-specific strategies.

## Data Availability

The original contributions presented in the study are included in the article/supplementary material, further inquiries can be directed to the corresponding author.
